# Scrofuloderma

**Published:** 1871-05

**Authors:** 


### Selections.

### SCROFULODERMA.

Scrofulous affections of the skin are easily recognized. We have had, however, during the last six years, a good deal of practical experience regarding strumous maladies of the skin—scrofula in all its forms being a very common disease in Belfast. Mr. Milton, in a former number of this journal,* offered some important suggestions on the present subject; following his example, we include under that name all strumous complaints found on the skin, no matter where they are situated, as ulcers superficial and deep, warty patches, enlarged glands, and also the indolent and dull red tubercular formations that tend to suppurate, eventually becoming covered with a thick scab, from beneath which oozes unhealthy pus, causing ulceration that

* *Journal of Cutaneous Medicine*, No 13, vol. iv.

proceeds, accompanied by slight loss of substance, the process ending by the formation of a scar.

Scrofuloderma verrucosum, which Dr. M'Call Anderson has dignified by the name of Lupus verrucosum,* is met with in strumous subjects, commencing by the development of small circumscribed, dusky, red, or violet-colored patches, often assuming a somewhat tuberculated form—healing, like lupus, in the centre, and spreading at the margins. These little tubercular spots are in many instances confluent, but more frequently isolated; finally warty growths appear on these elevations, of a dusky hue, due to a thickened condition of the cutaneous papillæ. The eruption is of a tedious and chronic nature. We lately had a young girl for eight months under treatment with this disease, situated upon the instep of left leg. The little patient's health had always been fair. The affection on the leg presented irregular rugous patches covered with warty excresences, separated from each other, with a tendency to ulceraticn around the margin of the disease.

During the year 1867, 824 cases of skin disease were admitted at the Belfast Hospital for Diseases of the Skin; of this number, scrofulous affection occurred to the extent of 12 per cent., at which average these cases have remained, occasionally increasing, however, especially in the Spring. Indeed, from the number of patients admitted in the early part of the year, one may say, with Shakespere (who refers to the "King's evil" being cured by royal touch):—

> "Strangely visited people,
> All swollen and ulcerous, pitiful to the eye—
> The mere despair of surgery—he cures,
> Hanging a golden stamp about their necks,
> Put on with holy prayer."

With regard to enlarged or strumous glands, we know that lymphatic glands enlarge by cell hyperplasy, and afterwards are prone to undergo cheesy degeneration, ending occasionally in an ulcer; by which we are to understand one determined by the inflammation and ulceration of a lymph gland enlarged by cell hyperplasia, and which has undergone degeneration. These ulcers are liable to occur about the region of the neck, in company with eruptions on mouth and cheeks, usually impetiginous, or with otorrhœa, discharge from mucous membrane of mouth and nose, conjunctivitis, etc. They are characterized by great obstinacy, and encroachment upon the connective tissue, and also by their serrated borders. In scrofula, it is the glandular

* *Journal of Cutaneous Medicine*, No. 1, vol. i.

system that is primarily affected, accompanied by engorgement of the capillary vessels and languid circulation, the blood being decidedly degenerated.

Dr. Sterl, of New York, has lately (*Medical Gazette*) written on the production of scrofula. He finds that, in scrofulosis, perfect oxygenation of the blood does not take place; for physiological experiments have proven that fibrin, so necessary in supplying muscular waste, is formed in the blood from albumen by the action of oxygen. He says:—

"The air of our city (New York) we know, is to a certain extent vitiated, and does not contain the requisite proportion of oxygen; but, in addition to this, our population is undoubtedly afflicted with an abnormal condition of the receptacles of this oxygen in the lungs; the blood cells, which, owing to the use of insufficient, unsuitable, or deleterious food, or from other causes, do not possess the vitality necessary to a healthy performance of their functions.

"The urine of scrofulous persons, and that of children, selected for obvious reasons, contains a superabundance of the salines, with an excess of hydrochloric, phosphoric, and lactic acids; not unfrequently oxalic acid.

"The perspiration has a decidedly acid reaction, and is of more than usually offensive odor.

"The secretions from mucous membranes are augmented, and tend to become excessively albuminous under inflammatory action.

"Lymph, too, exists in superabundance, owing to the inordinate activity of hypertrophied lymphatic vessels and glands, and, like the blood, it contains more than its natural proportion of albumen.

"The scrofulous diathesis I have always found most decidedly marked during and before the period of the first dentition."

Some French physicians divide the great class of scrofulides into benign and malignant; and, as symptoms of the first period of scrofula, recognize primary lesions, classified as exudative, erythematous, and papular. In the first-mentioned class, strophulus, impetigo, and sebaceous acne are contained, all forms of the latter being regarded as strumous. Amongst the erythematous.forms of scrofulderma, M. Bazin places chilblains, especially those accompanied b y deep-seated chronic inflammation of the subcutaneous tissue, a structure affected in preference by scrofula.

Tenacity, persistence, gradual extension to new tissue, participation of lymphatic glands and sub-cutaneous cellular tissue, ending in suppuration, are characters common to scrofuloderma, which, moreover, differs from other skin complaints in absence of itching, and usually of pain.

Cazenave looked on "malignant scrofulides" as manifestations of hereditary syphilis, being remarkable for their well-defined limits and tendency to relapse. "These eruptions are divided into three classes—ulcero-crustaceous, tuberculous, and erythematous. The crustaceous scrofulide contains two important varieties—inflammatory ulcerating and ulcerating with fibro-plastic formations. The first commences with tubercles or pustules simply inflammatory, which degenerate into ulcers that destroy surrounding soft parts, but are arrested by the bones. These ulcers cover themselves with thick green crusts, imbedded in the skin, and formed of concentric layers. After the crust has fallen and the ulcer healed, there remains a white irregular cicatrix, contracting the tissue like those of a burn, and adherent to the bone. In the second variety, the tubercles are fibro-plastic, caused by a proliferation of the cellular tissue, and the ulcers attack the bone as well as soft parts." The primitive element in the class of tubercular scrofulides is a fibro-plastic tubercle remaining stationary without ulceration on the cutaneous surface; however, cicatrices are produced, as if from an open ulcer, new fibrous tissue filling up the excavation caused by destruction of sub-cutaneous cellular tissue. The erythematous scrofulides of Bazin appear to be of an innocent nature. In some cases the subcutaneous tissue becomes œdematous, and of a pasty feel. There is no burning, itching, or pain, and eventually a white, irregular cicatrix appears in the centre of the patch, which gradually extends to the circumference. Malignant scrofulides are distinguished from cancer by the edges of the ulcers, which are undermined, instead of prominent; by the bottom, which does not present the hard, fleshy, granulations of cancer; and by absence of pain.

A few words on treatment, and we are done. Mr. Milton,* who, as a practical observer and trustworthy writer, is excelled by no one, places great faith in "plenty of purgatives." He believes that the patients get well as quickly, if not quicker, by this plan, than with iron, cod-liver oil, etc. A tablespoonful of old rum in a half-a-pint of new milk every morning, is, he thinks, a capital nutritive agent.

Dr. Laycock recommends the phosphates of lime, either pure or as ivory dust, in the various forms of struma, as well as in some syphilitic affections, on account of it supplying phospho-

* See Mr. Milton's paper, *Journal of Cutaneous Medicine*, No. 13.

rus to the nerve tissue. The local treatment is all-important: if there be much inflammation, poultices are required. Say we have in the person of a young lady an enlarged and suppurating gland on an exposed part, and which has been religiously poulticed for several days, how shall we best avoid a mark? Simply by applying a leech, and through the leech-bite, with a fine knife, such as is used for operations about the eye, make the necessary puncture—not incision. To prevent an enlarged gland from suppurating, we know of no remedy except nauseating doses of tartar emetic, and ice to the gland. It is not every one, however, who will permit of this treatment. When an enlarged gland is in an "indolent" state, it is a good plan to apply a fly-blister over it, which will either hasten suppuration or excite absorption; or Mr. Jordan's plan of blister near the enlarged gland, and application of a bag containing shot to enlargement, can be tried. If it be an open strumous ulcer, the iodide of lead ointment is a very excellent and well-known remedy; if, however, the discoloring of the skin is objected to, iodide of cadmium may be substituted. When there is much discharge, astringents at commencement may be necessary, as sugar of lead, tannin, etc. The country people often use an alcoholic decoction of fresh walnut leaves with which to bathe and apply to the open "sores," and it is said with advantage. Some excellent remarks are contained in a back number of the *Medical Circular*, on the treatment of the disease under notice, as well as in causing scars by meddlesome surgery in scrofulous glands. The scars do not depend, the writer says, on the strumous glands being opened, but on the time of doing so. If opened at all, they should be opened very early. A scrofulous gland, when it begins to suppurate, contains a substance like soft cheese; afterwards a thin ichor. If it has softened down before it begins to adhere to the integument, the method then to prevent scars will be to make a longitudinal incision before the integuments are spoiled, with a knife, and press out all the softened gland. The cavity will then granulate and heal, and nothing remain but a white line. If the gland be left to pursue its course, and, still worse, if poultices be applied—or if much inflamed, and tincture of iodine be used, the gland adheres to the integument, which becomes absorbed and destroyed; and after healing, the wound becomes puckered, arising from loss of substance: hence the scar. Empirical treatment of disease, and especially of scrofula, is often absurd. Heberden, in his *Commentaries* writes as follows:—"It has been an old dispute among physicians whether the empirical or rational method of curing disease was to be preferred. If by the empirical method be meant that which is founded on facts recorded by others or observed by ourselves, it must be allowed that by this means only has the practice of physic been established. Fact and repeated experiments have alone informed us that jalap will purge, and ipecacuanha vomit; that the poppy occasions sleep, and bark will cure an ague. If we examine the whole Materia Medica and the whole practice of physic, we shall not find one efficacious, simple, nor one established method of cure, which was discovered or ascertained by any other means."

The constitutional treatment of scrofulous affections may be dismissed in a few words: plenty of out-door exercise; residence, if possible, at the sea-coast; and, as regards medicinal agents, cod-liver oil, syrup of the iodide of iron; iodide of ammonium, iodide of potash; or, in some cases, Neligan's solution of arsenic, iodine, and iodide of potash. A good purgative, as recommended by Mr. Milton, containing calomel, is also of service, given about every seven days. In some cases, tincture of iodine, administered in sherry, answers very well—or used locally, five to eight drops being injected, with a hypodermic syringe, into an enlarged gland.

A few words will be found in our *Miscellaneous Memoranda* on cerealin, and the manner in which linseed and bran are used at the Ulster Institution for Deaf and Dumb, since 1862, with marked success, in diminishing the number of strumous complaints—to which, by the way, as we have shown in table of diseases* observed at all the English, Scotch, and Irish Asylums, deaf-mutes are very subject. For delicate children of a scrofulous diathesis, "wheat phosphates," which is really cerealin, should be given with milk, especially as pointed out by Dr. Klencke, of Leipzic, that the milk of stall-fed cows contains very little sugar, and that the butter and casein are diminished, whilst the albumen is often as high as 15 per cent. Moreover, stall-fed cattle are liable to become tuberculous, as testified by Dr. Carsewell.

Such are the principal views regarding the pathology and treatment of a very common disease.—*Journal of Cutaneous Medicine.*

* Observations on Medical and Sanitary Treatment of Deaf and Dumb. By H. S. Purdon, M.D. 1868.

---

A CURE FOR HEMORRHOIDS.—Calomel applied (*Pacific Med. and Surg. Jour.*) dry once or twice a day to tumid and tender hemorrhoids rarely fails to *cure* them in a few days.

